# Associations of plasma neurofilament light chain with cognition and neuroimaging measures in community-dwelling early old age men

**DOI:** 10.1186/s13195-024-01464-1

**Published:** 2024-04-25

**Authors:** Rongxiang Tang, Erik Buchholz, Anders M. Dale, Robert A. Rissman, Christine Fennema-Notestine, Nathan A. Gillespie, Donald J Hagler, Michael J. Lyons, Michael C. Neale, Matthew S. Panizzon, Olivia K. Puckett, Chandra A. Reynolds, Carol E. Franz, William S. Kremen, Jeremy A. Elman

**Affiliations:** 1 Department of Psychiatry, University of California San Diego, La Jolla, 92093 USA; 2 Center for Behavior Genetics of Aging, University of California San Diego, La Jolla, 92093 USA; 3 Department of Radiology, University of California San Diego, La Jolla, 92093 USA; 4 Department of Neurosciences, University of California San Diego, La Jolla, 92093 USA; 5 Department of Psychiatry, Virginia Institute for Psychiatric and Behavior Genetics, Virginia Commonwealth University, Richmond, VA 23284 USA; 6 QIMR Berghofer Medical Research Institute, Brisbane, QLD Australia; 7 Department of Psychological and Brain Sciences, Boston University, Boston, 02215 USA; 8 Department of Psychology and Neurosciences, University of Colorado Boulder, Boulder, 80309 USA

**Keywords:** Neurofilament light chain, White matter hyperintensity, Processing speed, Neurodegeneration, Blood-based biomarkers

## Abstract

**Background:**

Plasma neurofilament light chain (NfL) is a promising biomarker of neurodegeneration with potential clinical utility in monitoring the progression of neurodegenerative diseases. However, the cross-sectional associations of plasma NfL with measures of cognition and brain have been inconsistent in community-dwelling populations.

**Methods:**

We examined these associations in a large community-dwelling sample of early old age men (*N* = 969, mean age = 67.57 years, range = 61–73 years), who are either cognitively unimpaired (CU) or with mild cognitive impairment (MCI). Specifically, we investigated five cognitive domains (executive function, episodic memory, verbal fluency, processing speed, visual-spatial ability), as well as neuroimaging measures of gray and white matter.

**Results:**

After adjusting for age, health status, and young adult general cognitive ability, plasma NfL level was only significantly associated with processing speed and white matter hyperintensity (WMH) volume, but not with other cognitive or neuroimaging measures. The association with processing speed was driven by individuals with MCI, as it was not detected in CU individuals.

**Conclusions:**

These results suggest that in early old age men without dementia, plasma NfL does not appear to be sensitive to cross-sectional individual differences in most domains of cognition or neuroimaging measures of gray and white matter. The revealed plasma NfL associations were limited to WMH for all participants and processing speed only within the MCI cohort. Importantly, considering cognitive status in community-based samples will better inform the interpretation of the relationships of plasma NfL with cognition and brain and may help resolve mixed findings in the literature.

**Supplementary Information:**

The online version contains supplementary material available at 10.1186/s13195-024-01464-1.

## Background

Neurofilament light chain (NfL) has been researched as a non-specific biomarker of neurodegeneration in a wide range of neurological and neurodegenerative diseases such as Alzheimer’s disease (AD), frontotemporal dementia, and Parkinson disease [1–3]. As an axoskeletal protein that maintains myelinated large-caliber axons, NfL concentrations elevate in cerebrospinal fluid (CSF) and blood (i.e., plasma, serum) following neuroaxonal damages or degeneration [3, 4]. For instance, elevated CSF and blood-based NfL concentrations have been detected in individuals with AD and non-AD neurodegenerative diseases [1–5], as well as those undergoing normal aging process [6, 7]. Because blood-based NfL is less invasive and more cost-effective compared to neuroimaging and CSF measures of neurodegeneration, studies have evaluated blood-based NfL as a potential biomarker of neurodegeneration for monitoring and predicting neurodegenerative disease progression [1, 2, 8]. Specifically, research has examined blood-based NfL’s associations with multiple domains of cognition and neuroimaging measures of gray and white matter to better understand whether or not blood-based NfL shows specificity to certain cognitive domains and aspects of neurodegeneration. However, findings on the cross-sectional associations of blood-based NfL concentrations with cognition and neuroimaging markers of neurodegeneration have been mixed in community-dwelling individuals without a clinical diagnosis of dementia.

Several community-based studies in dementia-free middle-aged and older adults have shown that plasma or serum NfL level is associated with performance on the Mini Mental State Exam (MMSE) [6, 9, 10], as well as one or more specific cognitive domains including memory, language, attention, and executive function [10–13]. However, other studies in community-dwelling individuals found no reliable associations between plasma NfL and these cognitive domains [14, 15]. The inconsistencies might be attributed to differences in age ranges across the study samples (e.g., 30–66 years old vs. 55–90 years old) [10, 15], as there is evidence suggesting a stronger association between NfL and cognition in those greater than 50 years old relative to those who are much younger [10]. Moreover, differences in cognitive status among community-dwelling individuals may also affect the strength of associations, such that some associations were only detected in those with MCI but not cognitively unimpaired (CU) individuals [9]. Finally, some of the prior work only utilized a single task to measure a cognitive domain, which is not as reliable as those that used multiple tasks to tap a single domain [16, 17]. Nonetheless, there is still a lack of sufficiently large-scale studies to fully understand and resolve these inconsistent findings regarding the associations of blood-based NfL with multiple cognitive domains that are assessed by more than one task.

Similarly, studies on the cross-sectional associations between blood-based NfL and neuroimaging measures of neurodegeneration have yielded mixed results. For macrostructural measures of gray matter, there is evidence indicating that higher plasma NfL level is associated with lower cortical thickness and smaller hippocampal volume in community-based dementia-free individuals [11, 12, 18]. Conversely, there are studies that either failed to detect any association between plasma NfL, cortical thickness, and hippocampal volume [13, 19]. Similarly, a study that focused on cortical thickness of AD signature regions (i.e., regions vulnerable to AD pathology and neurodegeneration), also did not detect any association [13]. White matter hyperintensities (WMH), a marker of white matter damage, and diffusion-based metrics of white matter integrity (e.g., fractional anisotropy, mean diffusivity), have also been examined in relation to blood-based NfL. While one study found a cross-sectional association between higher plasma NfL level and lower white matter integrity in a large community-dwelling sample of individuals who are either CU or with MCI [13], two other studies in a large community-dwelling cohort with both CU individuals and those with MCI did not observe any association [11, 19]. Interestingly, another study examined these associations separately in CU and MCI groups, and found a significant association between plasma NfL and white matter integrity only in the MCI group [20]. These findings suggest that differences in sample composition with respect to cognitive status may explain the discrepant cross-sectional results. Finally, similar conflicting results have been reported for WMH, such that both no and moderate cross-sectional associations with blood-based NfL were found in community-dwelling samples without dementia [7, 11–13, 21].

These inconsistent cross-sectional findings of blood-based NfL’s associations with cognition and brain in community-dwelling samples undermines its potential clinical utility in the diagnosis or monitoring of neurodegeneration. As such, there is a need for additional large-scale community-based investigations before blood-based NfL is implemented in clinical settings. In the present study, we systematically examined the associations of plasma NfL with both cognition and neuroimaging measures in 969 early old age community-dwelling men (mean age = 67.57 years, range = 61–73 years) who were either CU or with MCI. Specifically, we investigated the associations of plasma NfL with five cognitive domains: executive function, episodic memory, verbal fluency, processing speed, and visual-spatial ability. Each of these cognitive domains was assessed by at least two tasks. Next, we examined plasma NfL’s associations with neuroimaging measures of gray and white matter commonly included in prior work (e.g., cortical thickness, volume), as well as a less examined measure of gray matter microstructural integrity – cortical mean diffusivity (MD) –which has been shown to be a sensitive marker of microstructural neurodegeneration that precedes atrophy in cortical thickness and volume [22]. Finally, we divided the sample into CU and MCI groups to explore whether the associations of plasma NfL with cognition and neuroimaging measures differ as a function of cognitive status, which may help resolve some of the mixed findings in community-dwelling samples and provide additional context to precisely characterize the nature of these associations.

## Methods

### Participants

A total of 969 community-dwelling men without dementia (mean age = 67.57 years, SD = 2.52; range = 61–73) from wave 3 of the Vietnam Era Twin Study of Aging (VETSA) were included in the present study [23]. VETSA is a multisite longitudinal study of aging and risk for MCI and AD beginning in middle age [23, 24]. All participants served in the U.S. military sometime between 1965 and 1975, with 80% reporting no combat exposure. They are similar to American men in their age cohort with respect to health, education, and lifestyle characteristics [25]. Participants traveled to the University of California, San Diego (UCSD) or Boston University (BU) to participate in the VETSA. However, MRI scans were only acquired at UCSD for wave 3. Informed consent was obtained from all participants and institutional review boards at both sites approved all protocols. Table 1 is a summary of the sample characteristics.

**Table 1:** Characteristics of the study sample

Characteristics	Mean (SD)					Group Differences
	Full Sample (*N* = 969)		CU (*N* = 815)	MCI (*N* = 142)		*P* values
Age	67.57 (2.52)	67.56 (2.51)			67.76 (2.52)	*p* > 0.05
Male (n)	969 (100%)	-			-	*-*
Years of Education	13.98 (2.09)	14.09 (2.11)			13.41 (1.92)	*p* < 0.001
Young Adult General Cognitive Ability (z-scores)*	0.31 (0.67)	0.31 (0.66)			0.32 (0.72)	*p* > 0.05
Health Status	1.80 (1.36)	1.80 (1.36)			1.82 (1.38)	*p* > 0.05
Plasma NfL (ng/L)	13.18 (6.88)	13.08 (6.84)			13.89 (7.18)	*p* > 0.05

Note: CU = Cognitively unimpaired; MCI = Mild cognitive impairment; Health status = Charlson Comorbidity Index; Non-residualized scores reported for plasma NfL; *The mean score for the full sample is equivalent to an IQ of approximately 105

### Plasma NfL collection and processing

Plasma specimens were collected under fasting conditions. Fasting began by 9:00 PM the night before testing, and specimens were acquired the following morning between 8:00 AM and 8:30 AM. Following the procedures of our prior work [26], NfL was assayed on a single-plex plate using the ultra-sensitive Simoa technology platform HD-1 (Simoa NFL Advantage Kit; Quanterix Corporation) by the USC Alzheimer’s Therapeutic Research Institute Biomarker Core (PI: Dr. Robert Rissman) [14]. All assays were performed according to the manufacturer’s instructions. The standard exclusion criteria included hemolysis and a coefficient of variation in plasma concentrations > 0.20.

### Cognitive domains

Factor scores for all five cognitive domains were derived from scores of the neuropsychological tasks. All task scores were adjusted for practice effects for returning participants [27]. Higher scores represent better cognitive performance. Below, we briefly described the tasks and conditions included in each cognitive domain.

#### Executive function

Measures from six well-established neuropsychological tasks were used to compute the executive function factor score: (1) Stroop [28] – interference condition, (2) Delis-Kaplan Executive Function System (D-KEFS) Trail Making Test [29] – switching condition, (3) Category Switching [29] – fluency switching condition, (4) Wechsler Memory Scale (WMS-III) Letter-Number Sequencing [30] – total number of correct trials, (5) Reading Span [31] – total number of correct words recalled, (6) Digit Span [30] – forward and backward conditions. For executive function, a confirmatory factor analysis was performed based on scores from six tasks to derive the factor score for each participant as described previously [32].

#### Episodic memory

The following tasks and conditions were used: (1) WMS-III Logical Memory (LM) subtest [30] with immediate and delayed recall conditions, (2) WMS-III Visual Reproduction (VR) subtest [30] with immediate and delayed recall conditions, and (3) the California Verbal Learning Test (CVLT-II) [33] with short- and long-delay free recall conditions. For the episodic memory factor score, a confirmatory factor analysis was performed using measures from three tasks to derive a factor for each participant as described previously [34].

#### Verbal fluency

The D-KEFS Verbal Fluency Test was used [29], which includes the phonemic fluency subtests (F, A, and S), two semantic fluency subtests (Animals and Boys’ Names), and a category switching subtest in which participants were instructed to alternate between naming fruits and items of furniture. For category switching subtest, the correct number of words generated aloud during the test was used as the score, rather than the correct number of switches. For the verbal fluency factor score, a confirmatory factor analysis was performed based on measures from two tests to derive the score for each participant as described previously [35].

#### Processing speed

For processing speed, there were three different tasks: (1) the D-KEFS Trails Making Test [29] with number sequencing and letter sequencing conditions, (2) Simple Reaction Time [36] with left hand and right hand conditions, (3) Stroop [28] with the word reading condition and the color naming condition. For the processing speed factor score, measures from three tests were used to derive the score for each participant using confirmatory factor analyses as described previously [37].

#### Visual-spatial ability

The following three tasks were used to index visual-spatial ability: (1) Card Rotations [38], (2) Hidden Fig. [39], and (3) Armed Forces Qualification Test (AFQT) [40] Box Folding subtest. For the visual-spatial ability factor score, measures from three tasks were used to derive a factor score for each participant from standardized factor scoring coefficients using exploratory factor analyses.

### MRI acquisition and processing

All acquisition parameters have been described in detail previously [41–43]. Briefly, T1-weighted images (sagittal 3D fast spoiled gradient echo (FSPGR), TE = 3.164 msec, TR = 8.084 msec), T2-weighted images (coronal 2D FRFSE-XL, TE = 94 msec, TR = 4600 msec), proton-density (PD)-weighted images (coronal, TE = min/full, TR = 3000 msec), and diffusion-weighted images (51 diffusion directions, b value = 1,000 s/mm2, integrated with a pair of b = 0 images with opposite phase encode polarity, TR = 9,700 ms, TE = 80–84 ms) were acquired on two General Electric (GE) Discovery MR750 3.0T scanners (GE Healthcare, Waukesha, WI, USA) at UCSD with an eight-channel phased array head coil.

For measures of cortical thickness and hippocampal volume, all MRI images were preprocessed at the UCSD Center for Multimodal Imaging Genetics as described previously [42, 44]. All raw and processed MRI images were visually inspected to exclude data with severe scanner artifacts or excessive head motion from subsequent analyses. T1-weighted images were processed as described previously [41, 42, 44]. Preprocessing steps included correction of distortion due to gradient nonlinearity, image intensity normalization, and rigid registration into standard orientation with 1 mm isotropic voxel size. Average cortical thickness, regional cortical thickness (for the signature below), and hippocampal volume were derived using FreeSurfer 6.0 (surfer.nmr.mgh.harvard.edu) software package.

Additionally, we derived an AD thickness/volume signature based on work by McEvoy and colleagues [45, 46]. This AD thickness/volume signature is a weighted average of cortical thickness in seven cortical regions (entorhinal cortex, middle temporal gyrus, bank of superior temporal sulcus, superior temporal gyrus, isthmus cingulate, medial orbitofrontal cortex, lateral orbitofrontal cortex) plus hippocampal volume. We regressed out the effects of age and scanner for each ROI, as well as estimated intracranial volume for the hippocampus. Standardized residuals of ROIs were then weighted accordingly and summed together to form the thickness/volume AD signature score.

For global volume of WMH, a multi-channel segmentation approach was used similar to our previous work [43]. This approach leverages complementary information from three volumes with different contrasts to increase measurement sensitivity while reducing the impact of MR acquisition noise. The measure used was the proportion of total white matter that had WMH, a ratio of global WMH to total white matter. Processing steps included standard alignment of the T1 (6 degrees-of-freedom, rigid transformation to an anterior/posterior commissure aligned space), registration of T2 and PD to T1 using a mutual information method [47] and intensity non-uniformity correction using N3 [48]. The tissue segmentation was performed using the statistical classification framework of the open source Insight Segmentation and Registration Toolkit (ITK) [49] leveraging Scott’s L2E method [50] to determine robust means and covariances, and in combination with anatomical considerations via ITK’s morphological operators [51]. This produced an automated segmentation including white matter, gray matter, CSF, and WMH. WMH included three categories within our T1-T2-PD feature space per Mahalanobis distance considerations [52], reflecting which primary tissue type was most similar to the abnormalities measured: WMH-white (properties more similar to white matter tissue), WMH-gray (properties more similar to gray matter), or WMH-fluid (properties more similar to CSF). Due to the dramatic curvature and convolutions of the most anterior and posterior aspects of the brain, common partial voluming of cortical gyri results in numerous false positive WMH voxel assignments. Therefore, we did not allow automated WMH assignments in the most posterior and anterior 20 mm of the brain. Additionally, since partial voluming of CSF and white matter along the edges of ventricles results in voxels with WMH-like signal, even in healthy individuals [53], our segmentation did not allow any voxels that touched (i.e., shared a common face, edge, or vertex with) a ventricular fluid voxel to be classified as WMH. The automated segmentations were visually reviewed and manually edited when necessary to correct misclassifications. To reduce human error, edits were restricted to only changing voxels between gray and WMH-gray (e.g., if a cortical voxel was mislabeled as WMH) or between fluid and WMH-fluid (e.g., if the central voxels of an WMH cluster were mislabeled as CSF). The automatically classified WMH-white voxels were never edited.

For regional white matter tract measures of integrity, diffusion-weighted images (DWI) were processed as described previously [54, 55]. Briefly, DWIs were corrected for eddy current distortion, head motion, B0 distortions, and gradient nonlinearity distortions. The b = 0 images were registered to T1 images using mutual information and then rigidly resampled into a standard orientation relative to the atlas-registered T1, with 2 mm isotropic resolution. Conventional diffusion tensor imaging (DTI) methods were used to model the diffusion tensor as an ellipsoid where eigenvalues λ1, λ2 and λ3 define the three primary axes. We computed Fractional anisotropy (FA) and mean diffusivity (MD) for 25 white matter fiber tracts using a probabilistic atlas (12 in each hemisphere plus corpus callosum) [56] and created a composite score based on the union of all fiber tracts.

Additionally, we computed MD for regional cortical gray matter (i.e., cortical MD) as described previously [55]. Briefly, white and gray matter voxels were labeled using the cortical surfaces and subcortical segmentations generated by FreeSurfer in processed T1-weighted image resolution (1 mm isotropic). Diffusion metrics were mapped to the cortical surface as previously described [55]. To minimize the effects of partial voluming and regional variations in cortical thickness, we take a weighted average of multiple samples across the cortical ribbon based on the proportion of gray matter at each sampling location. Vertex-wise values were then averaged within each cortical parcellation. Similarly, weighted averages of diffusion metrics within subcortical structures were based on voxel-wise proportions of gray matter within each subcortical segmentation [57]. Like the AD signature, a MD signature based on cortical MD (i.e., MD signature) of the same eight ROIs was computed as described previously [58].

### Young adult general cognitive ability

Participants were on average 20 years of age when they completed the Armed Forces Qualification Test (AFQT). The AFQT is a standardized, validated 100-item multiple-choice paper-and-pencil test of general cognitive ability [40]. It includes 4 components: vocabulary, arithmetic, spatial processing, and knowledge and reasoning about tools. AFQT percentile scores were probit transformed and z-scored for analysis. This test is highly correlated with other tests of general cognitive ability such as the Wechsler Adult Intelligence Scale (*r* = 0.84) [59].

### Health status

A modification of the Charlson Comorbidity Index was used [60]. One point was assigned for the presence of each of 15 different chronic medical conditions as described previously [42]. Higher scores indicate poorer health.

### Statistical analyses

All statistical analyses were performed using R version 4.1.2. Plasma NfL was adjusted for testing site and sample storage time using the *umx_residualize()* function from the umx R package. Residualized scores of plasma NfL were then log transformed to improve their distribution properties. All neuroimaging measures were adjusted for scanner differences. Hippocampal volume was additionally adjusted for intracranial volume (ICV). To examine the associations of plasma NfL with cognitive and brain measures, linear mixed models were performed using the lme4 package with plasma NfL as the independent variable. Age, health status, and young adult general cognitive ability (GCA) were included as covariates in all models unless otherwise noted. The inclusion of young adult GCA aimed to account for the effect of longstanding individual differences in cognitive ability on late midlife cognition and brain structures. In supplemental analyses, years of education was included as a covariate rather than young adult GCA to make the analyses more comparable to prior work, since most of the previous studies on plasma NfL do not have early adult GCA. Because there were twin pairs in our sample, we included twin pair ID as a random intercept in linear mixed models to account for correlated outcomes. *P*-values were calculated using Satterthwaite degrees of freedom approximation. Multiple comparison correction was applied using the Li and Ji approach for false discovery rate (FDR) control [61], which accounts for the correlated relationships among our dependent variables.

## Results

### Associations of plasma NfL with covariates

Consistent with prior work, higher plasma NfL level was significantly correlated with age (*r* = 0.13, *p* < 0.0001) and poorer health (*r* = 0.08, *p* = 0.014). The correlations of plasma NfL level with young adult GCA (*r* = 0.06, *p* = 0.059) and years of education (*r* = 0.06, *p* = 0.050) were not statistically significant.

### Plasma NfL and cognition

Among the five cognitive domains, plasma NfL was only associated with processing speed (β=-0.12, p_FDR−corrected_=0.0004), such that higher plasma NfL level was associated with slower processing speed (Table 2). Associations of plasma NfL with the other four cognitive domains were not significant either before or after multiple comparisons correction. In our supplementary analyses that covaried for years of education instead of young adult GCA, we also observed the same significant association with processing speed (β=-0.11, p_FDR−corrected_=0.004), but not with other cognitive domains either before or after multiple comparisons correction (ps > 0.05) (Table S1).

**Table 2:** Plasma NfL’s associations with cognition in the full sample

Model: Cognitive Domain ∼ Age + Health Status + Young Adult GCA + Plasma NfL				
*Outcomes*	*Beta Estimate*	*95% CI* *[upper, lower]*	*Uncorrected* *P value*	*FDR-corrected P value*
Executive Function	-0.05	[-0.11, 0.01]	0.073	0.073
Episodic Memory	-0.06	[-0.12, 0.01]	0.094	0.075
Verbal Fluency	-0.06	[-0.12, 0.00]	0.066	0.073
Processing Speed	**-0.12**	**[-0.18, -0.05]**	**< 0.0001**	**0.0004**
Visual-spatial Ability	-0.05	[-0.10, 0.00]	0.051	0.073

Note: *N* = 956, except visual-spatial ability (*N* = 955). Plasma NfL’s beta estimates, 95% CI, and *p* values are shown. Significant associations with cognitive domains are shown in bold

We repeated the aforementioned analyses with only cognitively unimpaired (CU) participants, but did not find any significant associations with cognitive domains after correcting for multiple comparisons (Table 3). Higher plasma NfL level was associated with poorer executive function, but this association did not survive correction for multiple comparisons (β=-0.06, p_uncorrected_=0.024, p_FDR-corrected_=0.096). Models covarying for years of education instead of GCA yielded similar results (Table S2). In the MCI group, plasma NfL level was only significantly associated with processing speed (β=-0.39, p_FDR-corrected_=0.0004) like it was in the full sample, but not with other cognitive domains (ps > 0.05). Covarying for years of education also did not change the results (β=-0.36, p_FDR-corrected_=0.0004) (Table S2).

**Table 3:** Plasma NfL’s associations with cognition in CU and MCI groups

Model: Cognitive Domain ∼ Age + Health Status + Young Adult GCA + Plasma NfL				
*Outcomes*	*Beta Estimate*	*95% CI* *[upper, lower]*	*Uncorrected* *P value*	*FDR-corrected P value*
*CU*				
Executive Function	**-0.06**	[-0.12, -0.01]	**0.024**	0.096
Episodic Memory	-0.03	[-0.09, 0.04]	0.425	0.340
Verbal Fluency	-0.05	[-0.12, 0.01]	0.123	0.123
Processing Speed	-0.05	[-0.12, 0.01]	0.111	0.123
Visual-spatial Ability	-0.05	[-0.10, 0.01]	0.085	0.123
*MCI*				
Executive Function	0.10	[-0.05, 0.24]	0.186	0.248
Episodic Memory	-0.15	[-0.31, 0.01]	0.066	0.132
Verbal Fluency	-0.01	[-0.16, 0.15]	0.913	0.730
Processing Speed	**-0.39**	**[-0.58, -0.19]**	**< 0.0001**	**0.0004**
Visual-spatial Ability	-0.01	[-0.14, 0.11]	0.836	0.730

Note: For CU, *N* = 814, except processing speed *N* = 813 and visual-spatial ability *N* = 812. For MCI, *N* = 142. Plasma NfL’s beta estimates, 95% CI, and *p* values are shown. Significant associations with cognitive domains are shown in bold

To determine if the association of plasma NfL with processing speed in the full sample was driven by MCI status, we tested for an interaction between MCI status and plasma NfL. Indeed, we found a significant interaction effect (β = -0.29, *p* < 0.0001, 95% CI [-0.44, -0.14]), indicating that the association was significantly stronger in the MCI group compared to the CU group.

### Plasma NfL and neuroimaging measures

Among all the neuroimaging measures, higher plasma NfL level was only significantly associated with greater WMH in the full sample both before and after FDR correction (β = 0.005, p_FDR−corrected_=0.035). As shown in Table 4, mean cortical thickness, hippocampal volume, cortical MD, FA and MD of white matter tracts, and AD and MD signatures were not associated with plasma NfL level (ps > 0.05, before and after FDR correction). Similar results were observed when covarying for years of education, such that plasma NfL level was only associated with the proportion of abnormal white matter volume both before and after FDR correction (β = 0.006, p_FDR−corrected_=0.015) (Table S3). When we repeated the analyses in CU and MCI groups separately, no significant associations between plasma NfL level and neuroimaging measures were detected (ps > 0.05, before and after FDR correction) (Tables S4-S5).

**Table 4:** Plasma NfL’s associations with brain structures in the full sample

Model: Brain Structures ∼ Age + Health Status + Young Adult GCA + Plasma NfL				
*Outcomes (Sample Size)*	*Beta Estimate*	*95% CI* *[upper, lower]*	*Uncorrected* *P value*	*FDR-corrected P value*
Mean Cortical Thickness (*N* = 451)	-0.001	[-0.01, 0.01]	0.841	0.565
Hippocampal Volume (*N* = 398)	-23.68	[-82.75, 35.38]	0.432	0.406
Cortical MD (*N* = 330)	0.000	[-0.005, 0.005]	0.904	0.565
FA of White Matter Tracts (*N* = 330)	-0.001	[-0.003, 0.001]	0.387	0.406
MD of White Matter Tracts (*N* = 330)	0.002	[-0.001, 0.005]	0.206	0.406
WMH (*N* = 414)	**0.005**	**[0.001, 0.009]**	**0.007**	**0.035**
AD thickness/volume signature (*N* = 382)	-0.125	[-0.477, 0.227]	0.487	0.406
MD signature (*N* = 278)	0.258	[-0.267, 0.783]	0.336	0.406

Note: N = Plasma NfL’s beta estimates, 95% CI, and *p* values are shown. Significant associations with cognitive domains are shown in bold. WMH = White Matter Hyperintensity, MD = Mean Diffusivity, FA = Fractional Anisotropy

## Discussion

Establishing reliable associations of plasma NfL with cognition and neuroimaging measures of neurodegeneration in community-based settings is important for understanding its utility in predicting risk and monitoring progress of neurodegenerative diseases. Existing cross-sectional associations have been largely inconsistent in community-dwelling dementia-free individuals. Leveraging a large-scale aging dataset with well-established neuropsychological tests encompassing five cognitive domains, we found that plasma NfL level was only significantly associated with processing speed in early old age men with MCI, but not in CU individuals. Similarly, there was no cross-sectional association between plasma NfL level with any of the neuroimaging measures of neurodegeneration, with the exception of an association with white matter pathology in the full sample. Together, our results suggest that plasma NfL may not be sensitive to cross-sectional differences in cognition or measures of gray matter neurodegeneration, but may capture some cross-sectional differences in white matter pathology among early old age dementia-free men at ages 61–73 years.

The overall lack of cross-sectional association between plasma NfL and various domains of cognition is in support of prior conflicting findings. The Mayo Clinic Study of Aging (MCSA) had a comparable sample size (*N* = 995) and a similar percentage of participants who were CU or with MCI as our sample [11]. That study showed significant plasma NfL associations with memory, attention, and language, which roughly corresponds to episodic memory, executive function, and verbal fluency in our study. In contrast, we only found a modest association with executive function in the CU group before FDR-correction. The inconsistency may be due to the age difference between the two samples. The median age of the MCSA sample is about 7 years older than VETSA, and the maximum age is about 10 years older: MCSA (median age [interquartile range, IQR]: 75.6 [67.0, 80.9]); VETSA (median age [IQR]: 68.3 [65.5, 69.8]). Because older age is consistently associated with higher plasma NfL level (i.e., neurodegeneration), it is possible that the amount of neurodegeneration in the relatively younger VETSA participants is not sufficient to exert any detectable effect on cognitive function. Indeed, the median level and IQR of plasma NfL in MCSA (median [IQR] 17.0 [11.8, 23.9]) is much higher than that of VETSA (median [IQR] 13.2 [9.0, 15.1]), suggesting a greater degree of neurodegeneration. This explanation is further corroborated by another study (*N* > 600) that included non-Hispanic white CU participants who are similar to VETSA participants in demographics, and found no reliable association with tasks of memory or language, but with tasks of executive function [14].

It is also worth highlighting that the modest effect sizes of these cross-sectional associations between plasma NfL and cognition might explain some of the observed inconsistencies in previous findings. A large population-based study (*N* ∼ 4500) found that plasma NfL was associated with memory, fluency, and executive function [13]. However, not only was the age range of the study much wider and older than our sample (71.9 ± 7.3 vs. 67.6 ± 2.5), but also the effect sizes of the observed associations in the two studies were small (β< ±0.13) and not significantly different from one another (i.e., overlapping confidence intervals of beta estimates). Thus, it is important to consider effect sizes rather than significance when evaluating the robustness of these associations and comparing the results across studies. Relatedly, although our CU and MCI groups were not significantly different in mean plasma NfL levels, we found a significant plasma NfL x MCI Status interaction effect for processing speed, such that the association was significantly in the MCI group. Moreover, the effect size of the MCI group was significantly stronger than those of the CU group and the full sample (i.e., medium vs. small effect). These findings suggest the need to first identify the cognitive status of each individual and then examine each cognitive group separately for a more precise characterization of the associations in community-dwelling populations.

For neuroimaging measures of gray and white matter, the lack of any associations with plasma NfL except WMH volume, was somewhat unexpected, since NfL has primarily been used as a biomarker of neurodegeneration. Nonetheless, our findings suggest no reliable associations between plasma NfL and macro- or micro-structural measures of neurodegeneration in gray matter or in regions vulnerable to AD. These findings are consistent with prior large-scale studies in community-dwelling samples (*N* > 400) that failed to detect an association with hippocampal volume [11, 13], mean cortical thickness [13, 18] or AD signature [13]. We additionally tested for an association with cortical MD, an earlier index of microstructural neurodegeneration of gray matter that precedes macrostructural neurodegeneration in thickness and volume [22]. However, plasma NfL was also not associated with this early marker of cortical microstructural neurodegeneration or our MD signature that focused on AD-vulnerable regions.

Considering the fact that NfL is most abundant in myelinated large-caliber axons found in white matter, it is possible, though speculative, that plasma NfL is not as sensitive to neurodegeneration in gray matter as it is to neurodegeneration in white matter. Alternatively, white matter degeneration might appear much earlier than gray matter atrophy in an early old age population and may thus be reflected earlier in plasma NfL levels [13]. A few studies have examined the associations between plasma NfL and white matter integrity (i.e., FA, MD). Although a large community-based study (*N* > 900, 67.7 ± 6.4 years old) found an association with MD but not FA [13], we did not detect an association with either measure in our relatively younger sample (*N* ∼ 430, 67.6 ± 2.5 years old). It is possible that this conflicting result may be due to age differences, as well as sex differences, as the other study did show that the associations were somewhat stronger in women than in men. However, additional work is needed to examine this possibility. Our only significant finding for white matter was the modest association of plasma NfL with WMH volume, a macrostructural measure of white matter damage or pathology. Previous studies have mostly detected a cross-sectional association between WMH volume and plasma NfL in large community-based samples (*N* ∼ 335 to 742) [7, 13, 62], with two other smaller sample size studies finding no association (*N* ∼ 192 to 341) [11, 12]. Although a direct comparison of effect sizes across the studies is impossible due to insufficient information, the inconsistent result regarding WMH volume is likely due to sample size differences.

Finally, our findings provide unique insights into the associations of plasma NfL with cognition and neuroimaging measures, in that we controlled for the effect of young adult general cognitive ability (GCA) in our models. This variable may be considered an index of an individual’s cognitive reserve prior to age-related cognitive decline [63]. Prior work from our group has shown that young adult GCA or cognitive reserve accounts for roughly 10% of variance in each of the five cognitive domains in later life, whereas years of education only accounts for less than 1% of variance [42]. Therefore, by accounting for the effect of young adult GCA we are able to more precisely examine associations of NfL with later life cognition and neuroimaging measures that are independent of longstanding individual differences in cognitive ability. In addition, it is possible that significant associations in other studies may have been driven largely by the older participants. In contrast, our study avoids this possibility on account of our narrow age range. Notwithstanding the strengths of our study, there are three limitations. First, because our sample only had male participants, we were not able to determine whether there are sex differences in the associations of plasma NfL with cognition and brain. However, our work provides valuable information on the strengths of these associations in male participants, which paves the way for future large-scale work focusing on sex differences. Second, most of our participants are largely non-Hispanic white, which means our results may not be generalizable to other racial/ethnic groups. Third, there may be potential selection bias in our longitudinal study sample, as participants with poor health are likely to drop out over the course of the study. However, this limitation is common to all research studies of aging, including those that begin enrolling at older ages (e.g., > 70 years old), which are subject to similar selection bias.

## Conclusions

The present study suggests that higher plasma NfL level is modestly associated with greater volume of macrostructural white matter pathology, but does not reliably associate with cognition, neuroimaging measures of gray matter (thickness, volume, MD), or DTI-based measures of white matter integrity in community-dwelling early old age men. The differential strength of associations between plasma NfL and domains of cognition in those with and without MCI, as this study found for processing speed, indicates that sample composition with respect to cognitive status may explain discrepant findings in the literature. Future studies assessing the utility of NfL for predicting and monitoring brain or cognitive decline will benefit from characterizing how these associations evolve with age or disease progression.

### Electronic supplementary material

Below is the link to the electronic supplementary material.

Supplementary Material 1

## Data Availability

The Vietnam Era Twin Study of Aging dataset is publicly available to qualified researchers, with restrictions. Information regarding data access can be found at http://www.vetsatwins.org/for-researchers/.

## Abbreviations

AD: Alzheimer’s disease
AFQT: Armed Forces Qualification Test
CU: Cognitively unimpaired
DTI: Diffusion tensor imaging
FA: Fractional anisotropy
GCA: General cognitive ability
MCI: Mild cognitive impairment
MD: Mean diffusivity
NfL: Neurofilament light chain
WMH: White matter hyperintensity
