# Supplementary material for: Associations of plasma neurofilament light chain with cognition and neuroimaging measures in community-dwelling early old age men
Source: Alzheimers Res Ther. 2024 Apr 25;16:90. doi: 10.1186/s13195-024-01464-1 (PMC11044425; doi:10.1186/s13195-024-01464-1)
Supplement: Supplementary file 1 — Supplementary Material 1 [file 13195_2024_1464_MOESM1_ESM.docx]

**Supplementary Materials**

**Supplementary Tables**

**Table S1.** Plasma NfL’s associations with cognition in the full sample.

| **Model: Cognitive Domain ~ Age + Health Status + Years of Education + Plasma NfL** | | | | |
| --- | --- | --- | --- | --- |
| *Outcomes* | *Beta Estimate* | *95% CI*  *[upper, lower]* | *Uncorrected*  *P value* | *FDR-corrected P value* |
| Executive Function | -0.05 | [-0.10, 0.01] | 0.114 | 0.128 |
| Episodic Memory | -0.05 | [-0.12, 0.02] | 0.128 | 0.128 |
| Verbal Fluency | -0.06 | [-0.12, 0.00] | 0.065 | 0.128 |
| Processing Speed | **-0.11** | [-0.17, -0.04] | **0.001** | **0.004** |
| Visual-spatial Ability | -0.04 | [-0.09, 0.02] | 0.208 | 0.166 |

Note: N=968, except visual-spatial ability N=967. Plasma NfL’s beta estimates, 95% CI, and p values are shown. Significant associations with cognitive domains are shown in bold.

**Table S2.** Plasma NfL’s associations with cognition in CU and MCI participants.

| **Model: Cognitive Domain ~ Age + Health Status + Years of Education + Plasma NfL** | | | | |
| --- | --- | --- | --- | --- |
| *Outcomes* | *Beta Estimate* | *95% CI*  *[upper, lower]* | *Uncorrected*  *P value* | *FDR-corrected P value* |
| *CU* |  |  |  |  |
| Executive Function | **-0.06** | [-0.12, -0.00] | **0.036** | 0.144 |
| Episodic Memory | -0.03 | [-0.09, 0.04] | 0.401 | 0.321 |
| Verbal Fluency | -0.06 | [-0.12, 0.01] | 0.102 | 0.165 |
| Processing Speed | -0.05 | [-0.12, 0.01] | 0.124 | 0.165 |
| Visual-spatial Ability | -0.04 | [-0.10, 0.02] | 0.191 | 0.191 |
| *MCI* |  |  |  |  |
| Executive Function | 0.14 | [-0.01, 0.29] | 0.077 | 0.154 |
| Episodic Memory | -0.11 | [-0.29, 0.08] | 0.279 | 0.372 |
| Verbal Fluency | 0.00 | [-0.15, 0.16] | 0.974 | 0.779 |
| Processing Speed | **-0.36** | [-0.56, -0.16] | **<0.0001** | **0.0004** |
| Visual-spatial Ability | 0.04 | [-0.11, 0.18] | 0.636 | 0.636 |

Note: For CU, N=814, except processing speed N=813 and visual-spatial ability N=812. For MCI, N=142. Plasma NfL’s beta estimates, 95% CI, and p values are shown. Significant associations with cognitive domains are shown in bold.

**Table S3.** Plasma NfL’s associations with brain structures in the full sample.

| **Model: Brain Structures ~ Age + Health Status + Years of Education + Plasma NfL** | | | | |
| --- | --- | --- | --- | --- |
| *Outcomes (Sample Size)* | *Beta Estimate* | *95% CI*  *[upper, lower]* | *Uncorrected*  *P value* | *FDR-corrected P value* |
| Mean Cortical Thickness (N=458) | -0.001 | [-0.01, 0.01] | 0.749 | 0.468 |
| Hippocampal Volume (N=458) | -21.40 | [-79.75, 36.95] | 0.473 | 0.402 |
| Cortical MD (N=334) | 0.001 | [-0.005, 0.006] | 0.647 | 0.462 |
| FA of White Matter Tracts (N=334) | -0.001 | [-0.003, 0.001] | 0.313 | 0.391 |
| MD of White Matter Tracts (N=334) | 0.003 | [-0.001, 0.006] | 0.104 | 0.260 |
| WMH (N=334) | **0.006** | [0.001, 0.009] | **0.003** | **0.015** |
| AD volume/thickness signature (N=388) | -0.126 | [-0.476, 0.225] | 0.482 | 0.402 |
| MD signature (N=281) | 0.323 | [-0.204, 0.851] | 0.231 | 0.385 |

Note: Plasma NfL’s beta estimates, 95% CI, and p values are shown. Significant associations with cognitive domains are shown in bold. WMH=White Matter Hyperintensities, MD=Mean Diffusivity, FA=Fractional Anisotropy.

**Table S4.** Plasma NfL’s associations with brain structures in CU and MCI participants, covarying for young adult general cognitive ability (GCA).

| **Model: Brain Structures ~ Age + Health Status + Young Adult GCA + Plasma NfL** | | | | |
| --- | --- | --- | --- | --- |
| *Outcomes* | *Beta Estimate* | *95% CI*  *[upper, lower]* | *Uncorrected*  *P value* | *FDR-corrected P value* |
| *CU* |  |  |  |  |
| Mean Cortical Thickness (N=390) | -0.003 | [-0.01, 0.01] | 0.458 | p>0.05 |
| Hippocampal Volume (N=344) | -24.09 | [-86.11, 37.92] | 0.447 | p>0.05 |
| Cortical MD (N=287) | -0.001 | [-0.006, 0.005] | 0.843 | p>0.05 |
| FA of White Matter Tracts (N=287) | -0.001 | [-0.003, 0.001] | 0.490 | p>0.05 |
| MD of White Matter Tracts (N=287) | 0.001 | [-0.002, 0.005] | 0.391 | p>0.05 |
| WMH (N=357) | 0.004 | [-0.000, 0.008] | 0.069 | p>0.05 |
| AD volume/thickness signature (N=331) | -0.086 | [-0.464, 0.292] | 0.657 | p>0.05 |
| MD signature (N=239) | 0.220 | [-0.312, 0.752] | 0.419 | p>0.05 |
| *MCI* |  |  |  |  |
| Mean Cortical Thickness (N=61) | 0.002 | [-0.020, 0.025] | 0.831 | p>0.05 |
| Hippocampal Volume (N=54) | 81.25 | [-111.55, 274.05] | 0.413 | p>0.05 |
| Cortical MD (N=43) | 0.008 | [-0.008, 0.024] | 0.352 | p>0.05 |
| FA of White Matter Tracts (N=43) | 0.000 | [-0.006, 0.007] | 0.908 | p>0.05 |
| MD of White Matter Tracts (N=43) | 0.003 | [-0.006, 0.012] | 0.495 | p>0.05 |
| WMH (N=57) | 0.008 | [-0.004, 0.021] | 0.200 | p>0.05 |
| AD volume/thickness signature (N=51) | -0.248 | [-1.394, 0.898] | 0.674 | p>0.05 |
| MD signature (N=39) | 0.959 | [0.535, 1.382] | 0.937 | p>0.05 |

Note: Plasma NfL’s beta estimates, 95% CI, and p values are shown. Significant associations with cognitive domains are shown in bold. WMH=White Matter Hyperintensities, MD=Mean Diffusivity, FA=Fractional Anisotropy.

**Table S5.** Plasma NfL’s associations with brain structures in CU and MCI participants, covarying for years of education.

| **Model: Brain Structures ~ Age + Health Status + Young Adult GCA + Plasma NfL** | | | | |
| --- | --- | --- | --- | --- |
| *Outcomes* | *Beta Estimate* | *95% CI*  *[upper, lower]* | *Uncorrected*  *P value* | *FDR-corrected P value* |
| *CU* |  |  |  |  |
| Mean Cortical Thickness (N=390) | -0.003 | [-0.01, 0.01] | 0.445 | p>0.05 |
| Hippocampal Volume (N=344) | -26.04 | [-88.12, 36.05] | 0.412 | p>0.05 |
| Cortical MD (N=287) | -0.001 | [-0.006, 0.005] | 0.837 | p>0.05 |
| FA of White Matter Tracts (N=287) | -0.001 | [-0.003, 0.001] | 0.495 | p>0.05 |
| MD of White Matter Tracts (N=287) | 0.002 | [-0.002, 0.005] | 0.357 | p>0.05 |
| WMH (N=357) | 0.004 | [-0.000, 0.008] | 0.057 | p>0.05 |
| AD volume/thickness signature (N=331) | -0.094 | [-0.474, 0.286] | 0.629 | p>0.05 |
| MD signature (N=239) | 0.219 | [-0.314, 0.752] | 0.422 | p>0.05 |
| *MCI* |  |  |  |  |
| Mean Cortical Thickness (N=61) | 0.009 | [-0.014, 0.033] | 0.428 | p>0.05 |
| Hippocampal Volume (N=54) | 70.11 | [-117.80, 258.02] | 0.468 | p>0.05 |
| Cortical MD (N=43) | 0.006 | [-0.010, 0.022] | 0.480 | p>0.05 |
| FA of White Matter Tracts (N=43) | 0.000 | [-0.006, 0.007] | 0.878 | p>0.05 |
| MD of White Matter Tracts (N=43) | 0.004 | [0.004, 0.005] | 0.369 | p>0.05 |
| WMH (N=57) | 0.009 | [-0.003, 0.021] | 0.154 | p>0.05 |
| AD volume/thickness signature (N=51) | 0.073 | [-1.017, 1.164] | 0.896 | p>0.05 |
| MD signature (N=39) | -0.170 | [-2.166, 1.825] | 0.863 | p>0.05 |

Note: Plasma NfL’s beta estimates, 95% CI, and p values are shown. Significant associations with cognitive domains are shown in bold. WMH=White Matter Hyperintensities, MD=Mean Diffusivity, FA=Fractional Anisotropy.
